# A Compressed Sensing-Based Wearable Sensor Network for Quantitative Assessment of Stroke Patients

**DOI:** 10.3390/s16020202

**Published:** 2016-02-05

**Authors:** Lei Yu, Daxi Xiong, Liquan Guo, Jiping Wang

**Affiliations:** 1 Jiangsu Key Laboratory of Medical Optics, Suzhou Institute of Biomedical Engineering and Technology, Chinese Academy of Sciences, No. 88, Keling Road, Suzhou, Jiangsu 215163, China; xiongdx@sibet.ac.cn (D.X.); guolq@sibet.ac.cn (L.G.); wangjp@sibet.ac.cn (J.W.); 2 University of Chinese Academy of Sciences, Beijing 100049, China

**Keywords:** compressed sensing, wearable sensor network, quantitative assessment, stroke, Brunnstrom stage classification

## Abstract

Clinical rehabilitation assessment is an important part of the therapy process because it is the premise for prescribing suitable rehabilitation interventions. However, the commonly used assessment scales have the following two drawbacks: (1) they are susceptible to subjective factors; (2) they only have several rating levels and are influenced by a ceiling effect, making it impossible to exactly detect any further improvement in the movement. Meanwhile, energy constraints are a primary design consideration in wearable sensor network systems since they are often battery-operated. Traditionally, for wearable sensor network systems that follow the Shannon/Nyquist sampling theorem, there are many data that need to be sampled and transmitted. This paper proposes a novel wearable sensor network system to monitor and quantitatively assess the upper limb motion function, based on compressed sensing technology. With the sparse representation model, less data is transmitted to the computer than with traditional systems. The experimental results show that the accelerometer signals of Bobath handshake and shoulder touch exercises can be compressed, and the length of the compressed signal is less than 1/3 of the raw signal length. More importantly, the reconstruction errors have no influence on the predictive accuracy of the Brunnstrom stage classification model. It also indicated that the proposed system can not only reduce the amount of data during the sampling and transmission processes, but also, the reconstructed accelerometer signals can be used for quantitative assessment without any loss of useful information.

## 1. Introduction

Stroke, also known as cerebrovascular insult (CVI), cerebrovascular accident (CVA), or brain attack, is when poor blood flow to the brain results in cell death. Between 1990 and 2010, the number of strokes which occurred each year decreased by approximately 10% in the developed world and increased by 10% in the developing world [1]. In 2013, stroke was the second most frequent cause of death after coronary artery disease, accounting for 6.4 million deaths (12% of the total) [2]. In China, stroke, with an annual mortality rate of approximately 157 per 100,000, has surpassed heart disease to become the leading cause of death and adult disability. In addition, China has 2.5 million new stroke cases each year and 7.5 million stroke survivors [3].

Considering the large population of stroke patients in China and the limited rehabilitation resources (rehabilitation centers, physicians, therapists), it is an inevitable trend for stroke patients to do rehabilitation training in home settings. Many previous research results have proven that, in comparison with inpatient care, home-based rehabilitation shows no difference in the effect on any of the outcomes [4,5]. Moreover, as there are fewer limitations on the time and space, patients can do rehabilitation training according to their own schedule in the home settings. However, due to the fact that there are no physicians or physiotherapists in these home settings to evaluate the motor function of stroke patients and adjust the prescribed training, how to precisely and automatically assess the motor function without the participation of physicians has become an important problem that needs to be resolved. Fortunately, the availability of wearable devices provides a potential approach to solve this problem.

With the development of the Internet of Things (IoT), wearable devices have been widely applied in the healthcare area. Considering sensor types, inertial measurement sensors such as accelerometers, gyroscopes, and magnetometers are used individually or together to monitor and analyze the motor functions of stroke patients [6,7]. In addition, some studies combine inertial measurement sensors with physiological sensors, like ECG, sEMG, *etc.* [8,9]. Based on the application scenarios, the following four categories can be distinguished: fall detection [10,11], physical activity monitoring [12,13,14], movement recognition [15,16,17] and quantitative assessment [18,19,20]. Particularly in the area of quantitative assessment for stroke patients, many valuable research results have been published. Patel *et al.* [21] proposed a Random Forests-based algorithm to estimate Functional Ability Scale (FAS) scores by using the signals of six accelerometers placed on the affected arm and the trunk. Based on the same dataset, Din *et al*. [20] established a Random Forests model to predict the Fugl-Meyer Assessment (FMA) scores. Uswatte *et al.* [22] have shown that just two accelerometers are adequate for assessing whether rehabilitation has an effect on arm function outside the laboratory. Previous work by our research group found that accelerometer data can be used to automatically classify the clinical Brunnstrom stages and some quantitative assessment indexes were designed to evaluate the motor function of stroke patients [23,24,25,26].

However, in our previous works, the accelerometer data were wirelessly transmitted from wearable devices to a receiver using the ZigBee protocol. Because the wearable devices are battery-operated, the battery life is inversely proportional to the amount of data transmitted. Hence, in order to extend the battery life and reduce the amount of data during the sampling and transmission processes, this paper proposes a novel wearable sensor network system based on compressed sensing technology. The main aim of this paper is to investigate whether the accelerometer signals collected during the training process of stroke patients could be compressed and whether the reconstruction errors have any influence on the quantitative assessment models. This paper is organized as follows: in Section 2, the wearable sensor network, compressed sensing technology and experiment protocols will be described. The experimental results and corresponding discussion will be described in Section 3. Finally, the contributions of this work and future work will be presented in Section 4.

## 2. Materials and Methods

### 2.1. Compressed Sensing-Based Wearable Sensor Network

**Figure 1:**
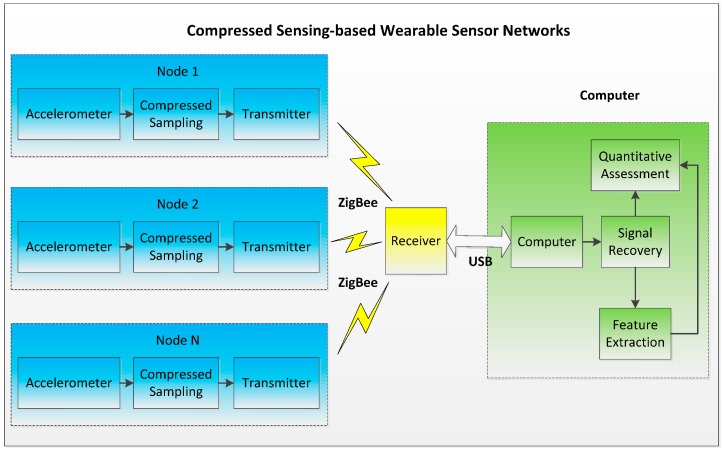
System structure of a compressed sensing-based wearable sensor network.

As shown in Figure 1, the compressed sensing-based wearable sensor network consists of three parts: an accelerometer sensor node, ZigBee wireless receiver, and computer. Compared with traditional accelerometer sensor nodes based on the Nyquist sampling theorem, the sensor node proposed in this paper has the advantage of “compressed sampling”, through which the sampling rate was reduced and the power consumption during the sampling and transmission processes were much lower than with the traditional method. The ZigBee wireless receiver was connected to the computer through a USB port and sends the compressed data to the computer. On the computer side, firstly, the accelerometer signal was reconstructed from the compressed data; secondly, those features that can represent the motor function of stroke patients were extracted using the time and frequency domain method; finally, through mapping the features were related to the clinical assessment outcomes given by physicians, and a quantitative assessment model was built.

### 2.2. Signal Recovery Algorithms

Compressed sensing (CS) [27,28,29] is a novel signal compression method that depends on the sparsity of signals for compression and reconstruction. The following Equation (1) describes the relationship of a fundamental noisy model:

(1) y=Φx+v

In this paper, $x\in {\mathbb{R}}^{N\times 1}$. is a part of a raw accelerometer signal, $y\in {\mathbb{R}}^{M\times 1}$ is the compressed data that will be wirelessly transmitted to a remote receiver via the ZigBee wireless protocol, and v can be omitted. $\Phi \in {\mathbb{R}}^{M\times N}\left(M\ll N\right)$ is a designed sensing matrix that linearly compresses **x**. Therefore, the model used in this paper is a noiseless model, expressed as:

(2) y=Φx

Assume the signal **x** was sparse under a certain orthogonal space $\Psi \in {\mathbb{R}}^{N\times N}$; that is, **x** can be represented by the following form:

(3) x=Ψθ

where **θ** = [*θ*_1_, *θ*_2_,…*θ_N_*]*^T^* was a *K*-sparse vector (K≪N), which means **θ** only has *K* non-zero elements.

In this paper, for ease of hardware implementation, the sparse Gaussian random matrix was adopted, where the value of every element was previously embedded into the microcontroller unit in the form of a lookup table.

Based on the framework proposed by Candes and Donoho [30], the principle of signal recovery was to solve the following $l_{1}$ norm optimization problem:

(4) $$\left\{ \begin{array}{l} \underset{\theta }{\min}{\left\|\theta \right\|}_{1}\\ s.t.y=\Phi \Psi \theta \end{array}\right.$$

In the past decades, many researchers have focused on this problem and proposed a series of recovery algorithms, such as matching pursuit (MP) [31], orthogonal matching pursuit (OMP) [32], basis pursuit (BP) [33], sparse Bayesian learning (SBL) [34] and so on. Considering the signal generally has block/group structure and there exists intra-block correlation among the elements within each block, Zhang *et al.* [35,36] proposed a new signal recovery framework called block SBL (BSBL) and the results showed that the performance was obviously better than that of other traditional methods. Hence, this paper chose the BSBL algorithm to reconstruct the accelerometer signal from compressed data. The basic principle of the BSBL framework is illustrated in the Appendix.

### 2.3. Extreme Learning Machine

**Figure 2:**
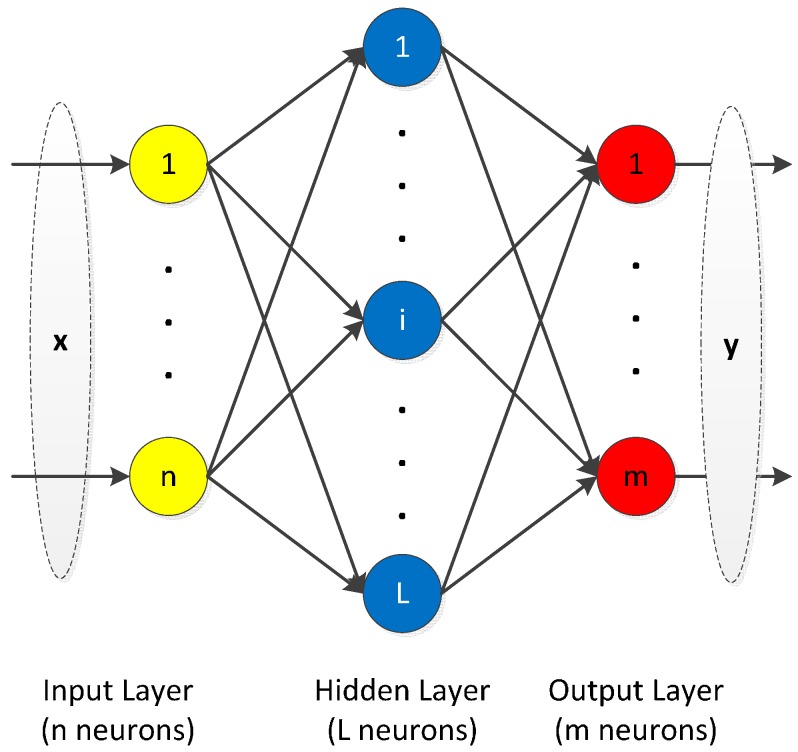
Structure of single layer feedforward network.

Extreme learning machine (ELM) was firstly proposed by Huang *et al.* [37], who applied it to the nonlinear mapping of a single layer feedforward network (SLFN). The SLFN structure is shown in Figure 2. In the following section, we will briefly introduce the principle of ELM.

Assume there are *N* samples $\left\{x_{i},y_{i}\right\}\left(i=1,2,\cdots ,N\right)$, where $x_{i}={\left[x_{i1},x_{i2},\cdots ,x_{in}\right]}^{T}$$\in {\mathbb{R}}^{n\times 1}$, $y_{i}={\left[y_{i1},y_{i2},\cdots ,y_{im}\right]}^{T}$$\in {\mathbb{R}}^{m\times 1}$, m and n are the size of the input and output vector, respectively. Equation (5) is the mathematically expression of a standard single layer feedforward networks (SLFNs), which has L hidden neurons with activation function g(x):

(5) Hβ=Y

where $Y={\left[y_{1},\cdots ,y_{N}\right]}^{T}$$\in {\mathbb{R}}^{N\times m}$, $\beta ={\left[\beta_{1},\cdots ,\beta_{L}\right]}^{T}$$\in {\mathbb{R}}^{L\times m}$ and:

(6) $$H\left(w_{1},\cdots ,w_{L},b_{1},\cdots ,b_{L},x_{1},\cdots ,x_{N}\right)=\left(\begin{array}{ccc} g\left(w_{1}x_{1}+b_{1}\right) & \ldots & g\left(w_{L}x_{1}+b_{1}\right)\\ \vdots & \ddots & \vdots \\ g\left(w_{1}x_{N}+b_{1}\right) & \cdots & g\left(w_{L}x_{N}+b_{1}\right) \end{array}\right)$$

where $w_{i}={\left[w_{i1},w_{i2},\cdots ,w_{in}\right]}^{T}$ is the weight vector connecting the input neurons and the ith hidden neuron, $\beta_{i}={\left[\beta_{i1},\beta_{i2},\cdots ,\beta_{im}\right]}^{T}$ is the weight vector connecting the output neurons and the ith hidden neuron, and $b_{i}$ is the threshold of the ith hidden neuron. 

The training process of an SLFN is simply the equivalent to finding a least-squares solution $\hat{\beta }$ of Equation (5):

(7) $$\left\|H\left(w_{1},\cdots ,w_{L},b_{1},\cdots ,b_{L}\right)\hat{\beta }-Y\right\|=\underset{\beta }{\min}\left\|H\left(w_{1},\cdots ,w_{L},b_{1},\cdots ,b_{L}\right)\beta -Y\right\|$$

According to the least-squares regression theory, the best solution of the linear system is:

(8) $$\hat{\beta }=H^{+}Y$$

where **H**^+^ is the pseudo inverse of **H**.

Compared with traditional backpropagation neural networks, ELM does not have to iteratively adjust the connecting weights and bias, as it maps the training process to a problem of solving a group of linear equations. Besides, in reference [37], Huang *et al*. have proved that given any small positive value ε>0 and activation function g:R→R which is infinitely differentiable in any interval, there exists $\tilde{N}\leq N$ such that for N arbitrary distinct samples, for any $w_{i}$ and $b_{i}$ randomly chosen, then with probability one, ‖Hβ−Y‖<ε.

In summary, the whole procedure of establishing an ELM model includes three steps, which are listed in Table 1.

**Table 1:** Flow of the ELM algorithm.

Step 1: Generate random input weight $w_{i}$ and bias $b_{i}$, $i=1,\cdots ,\tilde{N}$.
Step 2: Compute the output of neurons in hidden layer according to Equation (6).
Step 3: Compute the output weight β according to Equation (8).

### 2.4. Experiment Protocols

All the following experiments were approved by the Ethics Committee of Jiaxing 2nd Hospital, (Jiaxing, China). Twenty-three stroke patients and four physicians were selected to participate in these experiments. According to the Brunnstrom stage classification criteria, the 23 patients’ Brunnstrom stages ranged from II to V. However, considering that patients in stage I cannot move their upper limbs without extra assistance, patients in stage I were not selected for our experiments. Similarly, due to the fact that in China, the majority part of stage VI patients leave the hospital due to the high medical costs and limited rehabilitation resources, we used four physicians instead of stage VI patients to participate our experiments. 

Table 2 lists the general information of the 23 stroke patients, from which it can be clearly seen that there were 13 males and 10 females, with an age distribution range from 47 to 79. None of the patients had severe communication and cognitive problems. Before the data collection, all the experimental processes were demonstrated and the matters needing attention during the experiments were highlighted in advance by the physicians. 

**Table 2:** The general information of the 23 stroke patients.

Brunnstrom Stage Level	Patients	Sex (M/F)	Hemiplegic Side (Left/Right)	Limb Dominance (Left/Right)
II	2	0/2	2/0	0/2
III	10	5/5	6/4	2/8
IV	4	3/1	3/1	0/4
V	7	5/2	2/5	1/6

**Figure 3:**
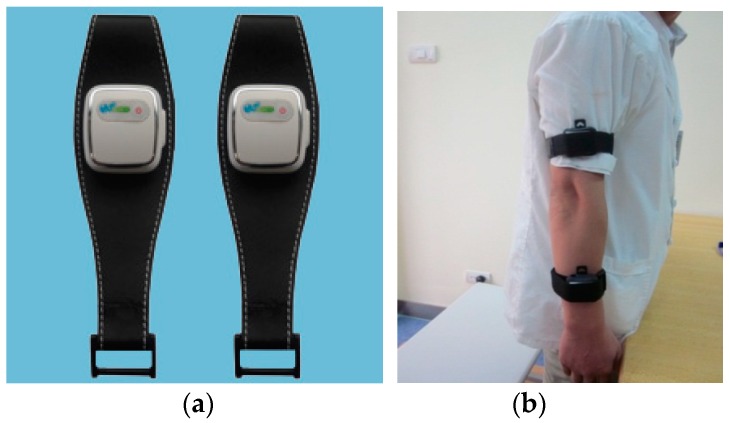
General view of (**a**) the accelerometer sensors and (**b**) the accelerometer sensor location.

The accelerometer sensors were placed on the geometric center of the arms, as shown in Figure 3. The distance between the accelerometer sensor on the forearm and dorsal stripes was 10 cm, while the distance between the accelerometer sensor on the upper arm and the epicondyus lateralis humeri was 8 cm.

Generally, in motion capturing and monitoring applications, reconstruction performance is often evaluated by comparing the reconstructed signals with raw ones through the mean square error (MSE). However, in this paper, the signal reconstruction was not the final goal, so the reconstructed accelerometer signals were further processed to establish an automatic Brunnstrom stage classification model. Due to the infidelity of the MSE for structured signals, it is difficult to analyze how the predictive accuracy of the quantitative assessment model is affected by the reconstruction errors measured by MSE. Hence, it is necessary to compare the predictive accuracy of Brunnstrom stage classification models, which are built based on the reconstructed and raw accelerometer signals, respectively. Consequently, to achieve our goal, all of the experiments were implemented in two actions: (1) Bobath handshake; (2) shoulder touch.

#### 2.4.1. Bobath Handshake

The purpose of this experiment is to validate the reconstruction performance of the BSBL algorithm during the Bobath handshake exercise. The whole process of the Bobath handshake exercise can be divided into the following three steps:

(1) Sit down on a chair, cross the hand and keep the thumb of the hemiplegic side on top.
(2) Straighten the upper extremities, lift above the head and hold for 3 s.
(3) Move the hands back to the initial position.

An additional movie file shows the Bobath handshake exercise in more detail (see Supplementary File 1).

#### 2.4.2. Shoulder Touch

Our previous studies have found that the accelerometer signals of the shoulder touch exercise can be applied to automatically classify the Brunnstrom stage for stroke patients. To investigate whether the reconstruction errors affect the predictive accuracy of the quantitative assessment model, the shoulder touch exercise was completed by every participant in order to collect the compressed accelerometer signals. The whole process of the shoulder touch exercise can be divided into the following four steps:

(1) Sit down on a chair, and naturally droop the upper limb of the hemiplegic side.
(2) Raise the upper limb of the hemiplegic side to the horizontal position.
(3) Move horizontally to the healthy side shoulder and hold for 5 s.
(4) Move back to the initial droop position and take a short break.

An additional movie file shows the shoulder touch exercise in more detail [see Supplementary File 2].

All patients were required to finish these exercises without extra assistance to reflect their true movement function. Before the data collection, they were asked to practice several times with the guidance of physicians so that they were familiar with the whole process. During the experimental processes, they were requested to repeat the shoulder touching exercise eight times.

The data sampling and management were implemented by using the Remote Rehabilitation Training and Assessment Software (RRTAS), which was developed by our group. The run environment of RRTAS is Windows 32 bit platform and .Net Framework 3.5 or above. An additional document file introduces RRTAS in more detail (see Supplementary File 3).

## 3. Results and Discussion

### 3.1. Accelerometer Signals Compress and Recovery

**Figure 4:**
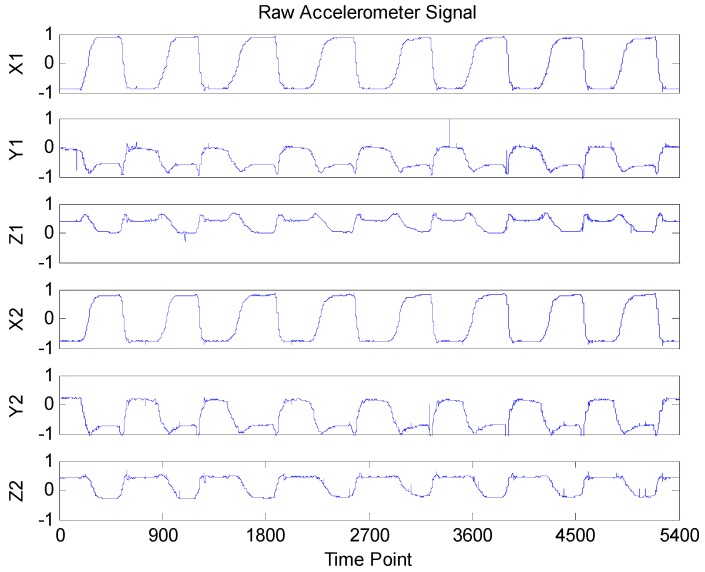
Raw accelerometer signals.

The raw accelerometer signal of the Bobath handshake exercise is shown in Figure 4, in which the axes X1, Y1 and Z1 are the signals of sensors placed on the forearm, while the axes X2, Y2 and Z2 are the signals of sensors placed on the upper arm. From Figure 4, we can see that during the Bobath handshake exercise, all six of the axes from two accelerometer sensors show period changes. However, 5400 raw sampling data in each axis need to be transmitted to the computer through the ZigBee wireless protocol during all eight Bobath handshake exercises. 

As mentioned above, by adjusting the dimension of sensing matrix Φ, the compression ratio (CR) can be changed as follows:

(9) $$CR=\frac{N-M}{N}=1-\frac{M}{N}$$

**Figure 5:**
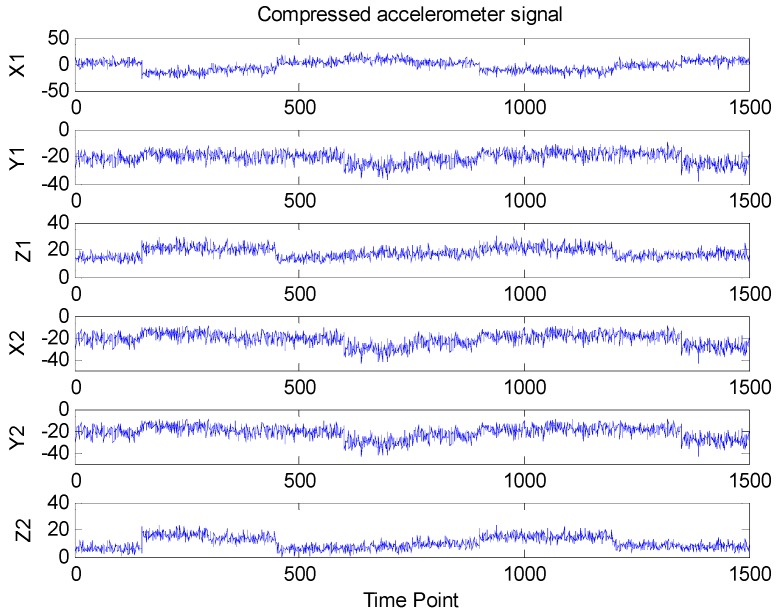
Compressed accelerometer signals.

Here, we first set the CR to 0.7222 to validate the reconstruction performance of the BSBL algorithm. This means that the raw accelerometer signal was compressed to 1500 sampling data. The corresponding compressed signal is shown in Figure 5, where it can be seen that the compressed signal looks very different from the raw accelerometer signal due to the randomness of the sensing matrix Φ. This characteristic indicates that compressed sensing can not only compress the raw signal into a lower dimension compressed signal, but also can encrypt the raw signal so that the privacy of the patients is protected. 

**Figure 6:**
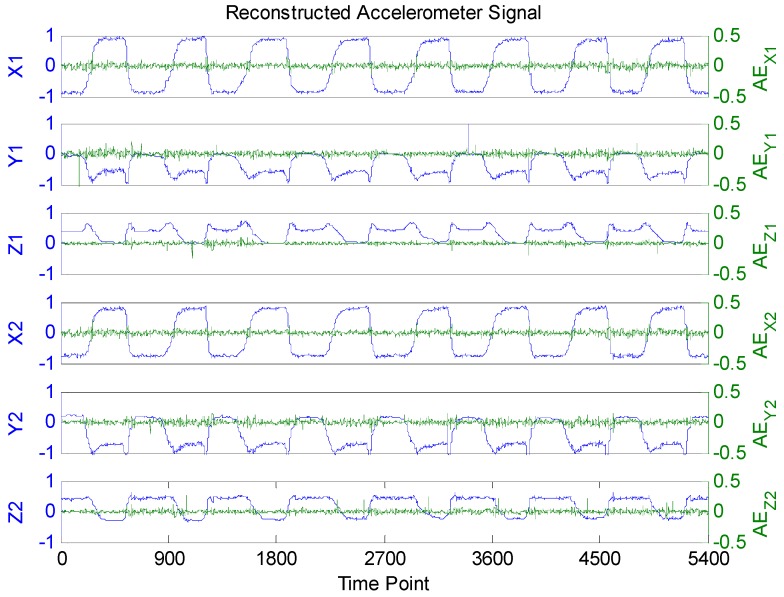
Reconstructed accelerometer signals and absolute errors (AE).

From Figure 4, it is obvious that the raw accelerometer signal is far from sparse and periodic. This creates a huge difficulty for traditional CS recovery algorithms to reconstruct the raw accelerometer signals. Fortunately, the BSBL recovery framework proposed by Zhang [35,36] significantly outperforms tradition CS algorithms thanks to its ability to explore and exploit an intra-block correlation in signals. When the prior block partition is given, the whole raw accelerometer signal is divided into 30 blocks of equal size (each block contains 5400/30 = 180 sampling data). The reconstructed accelerometer signals are shown in Figure 6. To validate the recovery performance of BSBL algorithm, we also reconstructed the compressed signals with BP and OMP algorithms, and the correlation coefficients of the reconstructed and raw signals of each algorithm are all listed in Table 3. It is clear that by using the BSBL algorithm, the correlation coefficients of the reconstructed and raw signals were higher than with the other two methods, which suggests that the BSBL algorithm is very effective for accelerometer signal recovery.

**Table 3:** Signal recovery results of BSBL, BP and OMP algorithms.

Methods	Correlation Coefficients between Reconstructed and Raw Signals					
	X1	Y1	Z1	X2	Y2	Z2
BSBL	**0.9991**	**0.9957**	**0.9965**	**0.9991**	**0.9979**	**0.9969**
BP	0.8762	0.8814	0.8729	0.8651	0.8964	0.9015
OMP	0.9356	0.9521	0.9188	0.9672	0.9248	0.9366

### 3.2. Effects of Block Size and CR on Recovery Performance

In this section, we will discuss the effects of block size and compression ratio on the quality of signals reconstructed from raw accelerometer signals. Here, we chose the signal to noise ratio (SNR) as the evaluation index to analyze the reconstruction error. The definition of SNR is shown in Equation (10):

(10) $$SNR=20\mathrm{lg}\frac{{∥x∥}_{2}^{2}}{{∥x-\overset{\wedge }{x}∥}_{2}^{2}}$$

where x and $\overset{\wedge }{x}$ are the raw and reconstructed signals, respectively. A higher SNR means a smaller reconstruction error.

**Figure 7:**
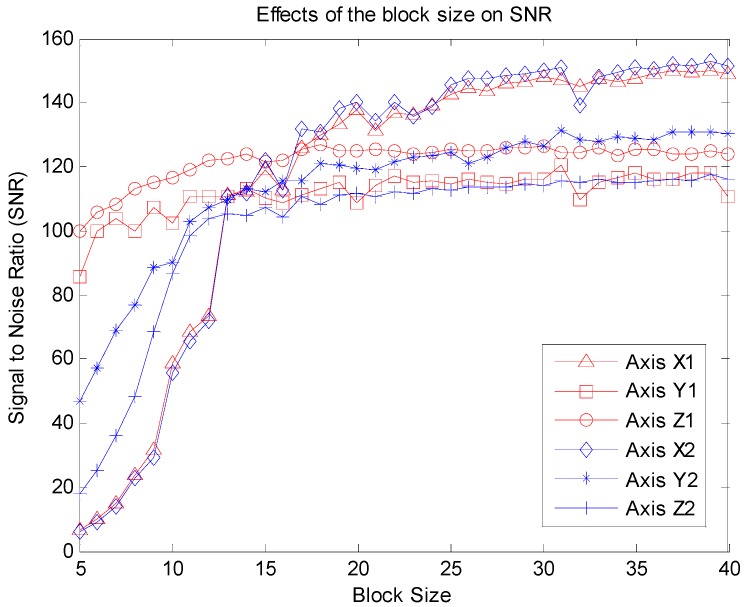
Effects of the block size on SNR.

The effects of block size on SNR are shown in Figure 7. It is clear that the trends of all six axes are same; that is, as the block size increases, the SNR also increases. In particular when the block size changes from 5 to 20, the corresponding SNR increases quickly. This is due to the fact that the sampling rate of the raw accelerometer sensor is 50 Hz, and for stroke patients the duration of the Bobath handshake exercise is about 12 s. Hence, if the block size is too small (for example, a block size less than 10), the signals in each block are almost unchanged because the duration of each block is very short. Consequently, all of the intra-block correlations of each block are close to 1 and the variances are close to 0. 

Although the reconstruction performance is affected by the block size in the block partition, this does not limit its application to the wearable sensor network. Figure 7 also shows that a wide range of the block size (for example, from 20 to 40) can lead to satisfying results.

**Figure 8:**
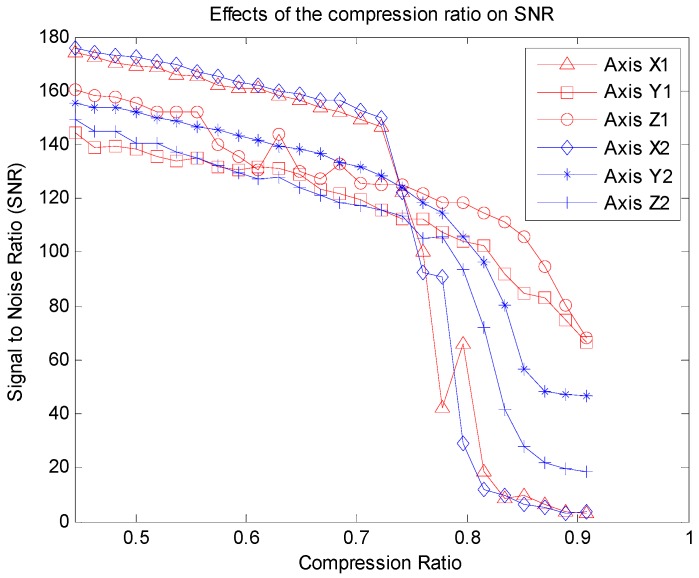
Effects of the compression ratio on SNR.

Figure 8 illustrates the effects of CR on SNR, from which we can see that when the CR increases, the SNR decreases, especially when the CR is higher than 0.75. That is to say, if the CR does not exceed 0.75 (the length of compressed signal cannot be less than 1350), a satisfactory quality reconstructed signal can be achieved. 

**Figure 9:**
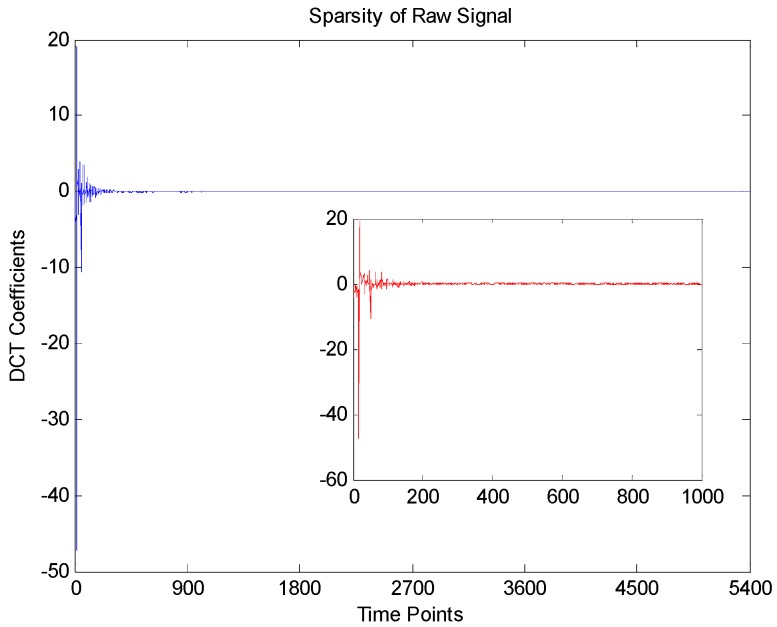
Sparsity of raw accelerometer signal (axis X1).

According to compressed sensing theory, the relationship between *M* and *N* should satisfy the following inequality:

(11) $$M>K\log\left(\frac{N}{K}\right)$$

where *K* is the sparsity of the raw signal. Now we validate the theoretical CR of the accelerometer signals. Figure 9 shows the sparsity discrete cosine transform (DCT) coefficients of the raw accelerometer signal. Clearly, only a few DCT coefficients (*K* ≈ 600) have large amplitudes, while the majority of coefficients have small amplitudes. Hence, the theoretical maximum CR should be:

(12) $$CR_{\max}=1-\frac{M_{\min}}{N}\approx 1-\frac{600\times \log\left(5400/600\right)}{5400}\approx 0.7559$$

This result is essentially the same as Figure 8, which means that the more sparser the raw signal is, the higher CR can be achieved.

### 3.3. Effects of Compressed Sensing on Quantitative Assessment Model

In the previous experiment, we showed that the accelerometer signals of rehabilitation exercises can be compressed and precisely reconstructed. Another question is whether the reconstructed signals affect the predictive accuracy of the quantitative assessment model. To answer this, we carried out the following experiment: as mentioned above, there are many assessment scales in clinical settings to evaluate the movement function of stroke patients, such as the Fugl-Meyer assessment scale, Functional ability scale, Brunnstrom stage classification, Action Research Arm Test, and so on. Compared with other assessment scales, the Brunnstrom stage classification tool only has six levels, hence it has the advantage of being easy to use and time saving. In our previous work [26], we have proven that the Brunnstrom stage of stroke patients can be automatically classified through the shoulder touch exercise by using the ELM method. 

**Figure 10:**
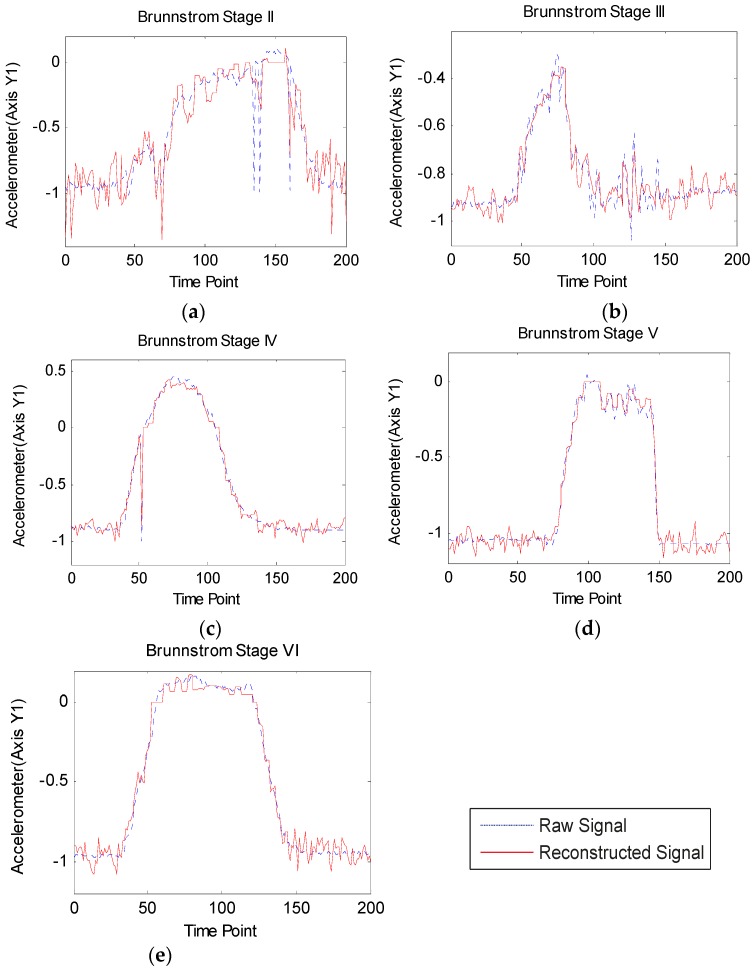
(**a**)–(**e**) Reconstructed and raw accelerometer signals from Brunnstrom stages II to VI.

Due to space limitations, here we present only the reconstructed and raw axis Y1 accelerometer signals ranges from Brunnstrom stage II to VI in Figure 10. It is clear that as the Brunnstrom stage increases, the reconstruction error decreases. This is because the lower the Brunnstrom stage, the weaker the stroke patient’s motor function, and he or she cannot finish the shoulder touch exercise freely. Additionally, with the Brunnstrom stage decreases, the smoothness of accelerometer signals becomes worse, which brings about difficulty in signal recovery. 

To investigate the effects of reconstruction error on the predictive accuracy of the Brunnstrom stage classification model, we established the quantitative assessment model with the reconstructed and raw accelerometer signals, respectively. To ensure the consistency of our comparison, the feature extraction and ELM parameters were all set to be the same. More specifically, 12 features including root mean square (RMS), amplitude (AMP) from three axes of two accelerometer sensors were extracted and set as the input of ELM model, the output of the ELM model was the Brunnstrom stage level, and the number of hidden neurons was set to 30. The training set contains 152 samples and the testing set contains 38 samples.

**Figure 11:**
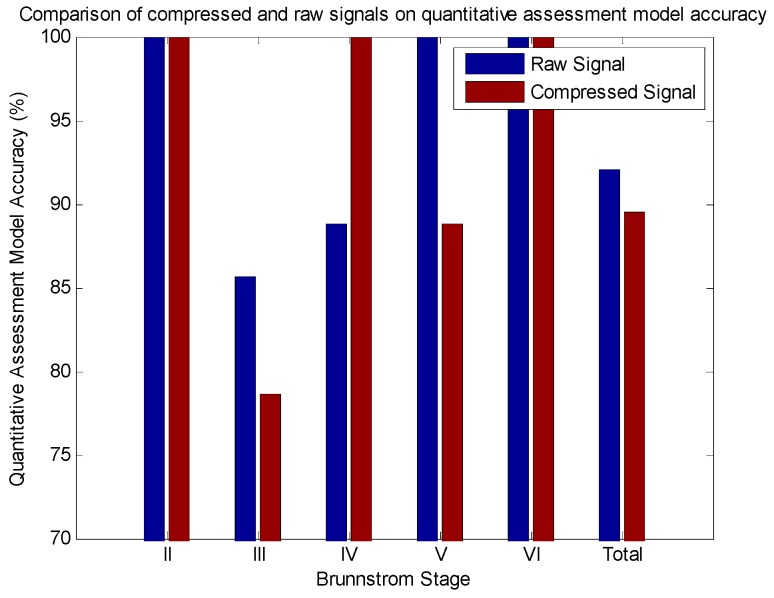
Comparison of compressed and raw signals on quantitative assessment model accuracy.

The results are shown in Figure 11 and Table 4, from which it can be seen that of the total 38 samples in the testing set, there were 34 samples whose Brunnstrom stages were accurately predicted by using the reconstructed signal, while 35 samples were accurately predicted using the raw signal, which indicated that the reconstruction error alone has no influence on the accuracy of the quantitative assessment model. Actually, the analysis of variance (ANOVA) result, listed in Table 5, indicates that there is no statistically significant difference between the predictive accuracy of the raw and reconstructed Brunnstrom stage classification model. Consequently, we can conclude that the reconstruction error has no influence on the establishment of a quantitative assessment model.

**Table 4:** Comparison of compressed and raw signals on quantitative assessment model accuracy.

Brunnstrom Stage	Samples in Testing Set	Predictive Accuracy	
		Raw Model	Compressed Sensing Model
II	2	100 (2/2)	100 (2/2)
III	14	85.7 (12/14)	78.6 (11/14)
IV	9	88.8 (8/9)	100 (9/9)
V	9	100 (9/9)	88.8 (8/9)
VI	4	100 (4/4)	100 (4/4)
Total	38	92.1 (35/38)	89.5 (34/38)

**Table 5:** Analysis of variance results.

Source	Sum of Squares	df	Mean Square	F	Prob > F
Between Groups	0.0132	1	0.01316	0.01	0.9205
Within Groups	96.9737	74	1.31046		
Total	96.9868	75			

## 4. Conclusions

To reduce the amount of data in a wearable sensor network during the sampling and transmission processes, this paper proposes a novel wearable sensor network based on compressed sensing technology, and applied it to the monitoring and quantitative assessment of stroke patients’ upper limb motor function. The experimental results showed that the proposed method can not only compress and precisely reconstruct the raw accelerometer signals, but can also apply the reconstructed signals to establish the Brunnstrom stage automatic classification model. It also indicated that the proposed system can provide a theoretical basis for individualized and remote rehabilitation. However, considering the fact that the clinical experiment only recruited 23 stroke patients, which is too small a number to verify the statistical reliability and validity of the proposed quantitative assessment model, in the future, we will gather more clinical data and improve the generalization performance of the model. Additionally, in this paper, the compressed signals need to be reconstructed on the computer side before the analysis process. In the future, we will try to investigate whether the analysis process can be directly implemented in the compressed domain. If yes, this could reduce the off-line computation burden and make the “on-node” analysis more possible.

## Supplementary Materials

Additional File 1: Bobath handshake exercise (Movies, MP4 format), Additional File 2: Shoulder touch exercise (Movies, MP4 format), Additional File 3: Introduction of Remote Rehabilitation Training and Assessment Software.

## Abbreviations

The following abbreviations are used in this manuscript:

CS: Compressed Sensing
WSN: Wearable Sensor Network
UE: Upper Extremity
WMFT: Wolf Motor Function Test
FAS: Functional Ability Scale
RIP: Restricted Isometry Property
MP: Matching Pursuit
OMP: Orthogonal Matching Pursuit
BP: Basis Pursuit
SBL: Sparse Bayesian Learning
BSBL: Block Sparse Bayesian Learning
CR: Compression Ratio
SNR: Signal to Noise Ratio
ELM: Extreme Learning Machine
DCT: Discrete Cosine Transform
RRTAS: Remote Rehabilitation Training and Assessment Software

## Appendix

Without loss of generality, assume x can be divided into g blocks, *i.e.*:

$$x={\left[\underset{x_{1}^{T}}{\underset{︸}{x_{1},\cdots ,x_{d_{1}}}},\cdots ,\underset{x_{g}^{T}}{\underset{︸}{x_{d_{g-1}+1},\cdots ,x_{d_{g}}}}\right]}^{T}$$

where $d_{i}\left(\forall i\right)$ are not necessarily identical. Among the g blocks, only k(k≪g) blocks are nonzero but their locations are unknown.

Each block $x_{i}\in$${\mathbb{R}}^{d_{i}\times 1}$ is assumed to satisfy a parameterized multivariate Gaussian distribution:

$$p\left(x_{i};\gamma_{i},B_{i}\right)\sim N\left(0,\gamma_{i}B_{i}\right),i=1,\cdots ,g$$

where $\gamma_{i}$ is a non-negative parameter controlling the block-sparsity of x. When $\gamma_{i}=0$, the i^th^ block becomes zero. $B_{i}\in$${\mathbb{R}}^{d_{i}\times d_{i}}$ is a positive definite matrix, capturing the correlation structure of the i^th^ block. Under the assumption that blocks are mutually uncorrelated, the prior of **x** is:

$$p\left(x;{\left\{\gamma_{i},B_{i}\right\}}_{i}\right)\sim N\left(0,\Sigma_{0}\right)$$

where $\Sigma_{0}\text{=diag}\left\{\gamma_{1}B_{1},\cdots ,\cdots \gamma_{g}B_{g}\right\}$. Assume the noise vector satisfies p(v;λ)~N(0,λI), where λ is a positive scalar. Therefore the posterior of **x** is given by:

$$p\left(x|y;\lambda ,{\left\{\gamma_{i},B_{i}\right\}}_{i=1}^{g}\right)=N\left(\mu_{x},\Sigma_{x}\right)$$

with:

$$\mu_{x}=\Sigma_{0}\Phi^{T}{\left(\lambda I+\Phi \Sigma_{0}\Phi^{T}\right)}^{-1}y$$

$$\Sigma_{x}={\left(\Sigma_{0}^{-1}+\frac{1}{\lambda }\Phi^{T}\Phi \right)}^{-1}$$

Once the parameters $\lambda ,{\left\{\gamma_{i},B_{i}\right\}}_{i=1}^{g}$ are estimated, the Maximum-*a-Posteriori* (MAP) estimate of x, denoted by $\overset{\wedge }{x}$, can be directly obtained from the mean of the posterior, *i.e.*:

$$\overset{\wedge }{x}\leftarrow \Sigma_{0}\Phi^{T}{\left(\lambda I+\Phi \Sigma_{0}\Phi^{T}\right)}^{-1}y$$

The parameters can be estimated by a Type II maximum likelihood procedure. This is equivalent to minimizing the following cost function:

$$L\left(\theta \right)≜\begin{array}{l} -2\log\int p\left(y|x;\lambda \right)p\left(x;{\left\{\gamma_{i},B_{i}\right\}}_{i}\right)dx\\ =\log\left|\lambda I+\Phi \Sigma_{0}\Phi^{T}\right|+y^{T}{\left(\lambda I+\Phi \Sigma_{0}\Phi^{T}\right)}^{-1}y \end{array}$$

where θ denotes all the parameters, *i.e.*, θ≜$\left\{\lambda ，{\left\{\gamma_{i},B_{i}\right\}}_{i=1}^{g}\right\}$.
