# Supplementary material for: A Compressed Sensing-Based Wearable Sensor Network for Quantitative Assessment of Stroke Patients
Source: Sensors (Basel). 2016 Feb 5;16(2):202. doi: 10.3390/s16020202 (PMC4801578; doi:10.3390/s16020202)
Supplement: Supplementary file 1 [file sensors-16-00202-s001.zip › Additional files/Introduction of Remote Rehabilitation Training and Assessment Software.pdf]

Introduction of Remote Rehabilitation Training and Assessment Software

- **Author: Lei Yu**, Suzhou Institute of Biomedical Engineering and Technology, Chinese Academy of Sciences (E-mail: yul@sibet.ac.cn; yuthreestone@gmail.com)
- **Run environment:** Windows 32 bit platform, .Net Framework 3.5 above
- **Develop environment:** Microsoft Visual Studio 2010 (C#)
- This soft have the following several functions:
  - Manage the personal information and individual training prescriptions through connect to MySQL database
  - Provide standard videos of 10 clinical common exercises (1. **Bobath handshake**; 2. Elbow extension; 3. Shoulder abduction; 4. Elbow flexion; 5. **Shoulder touch**; 6. Pronation-supination; 7. Wrist pressing; 8. Finger touch; 9. Cylinder grip; 10. Ball catch)
  - Real time sampling and plot of accelerometer signals (only feature axis is plotted)
  - Data save to disk (.bin format) and write to MySQL database
  - Automatic Brunnstrom stage classification and Scoring (centesimal system)

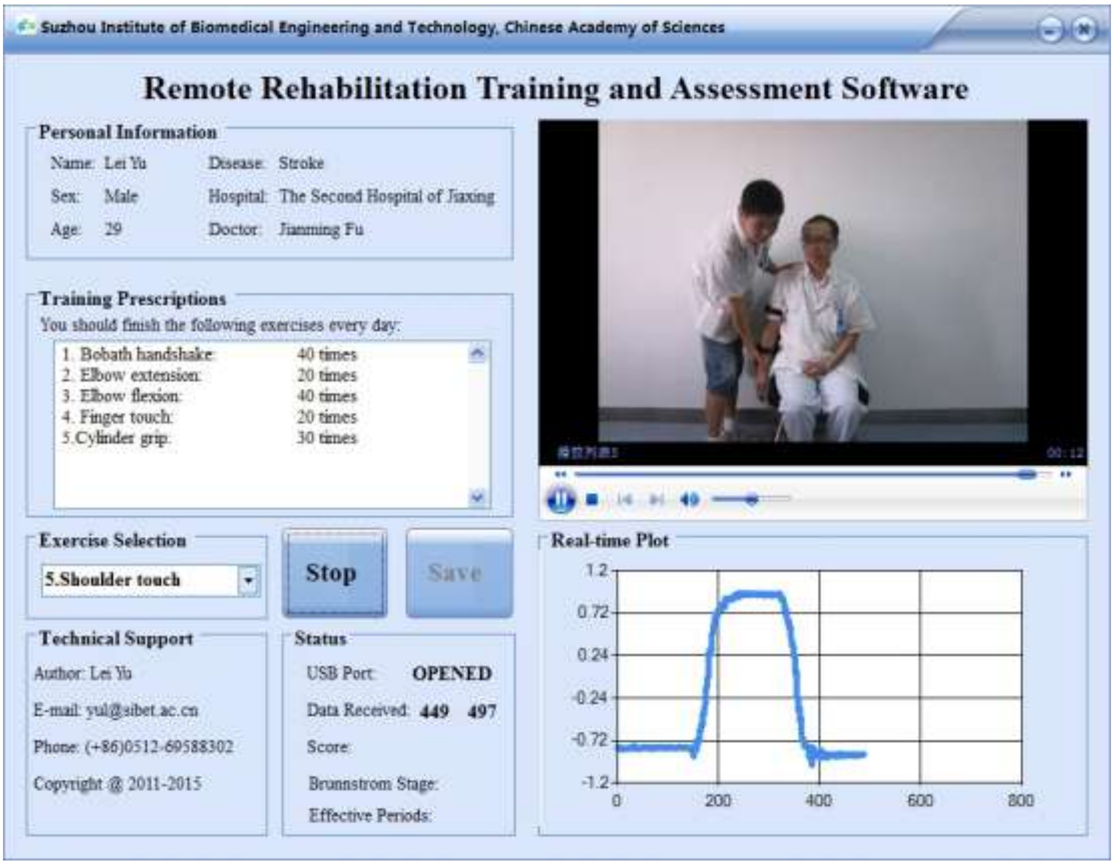

Figure 1 Real time sampling and plot

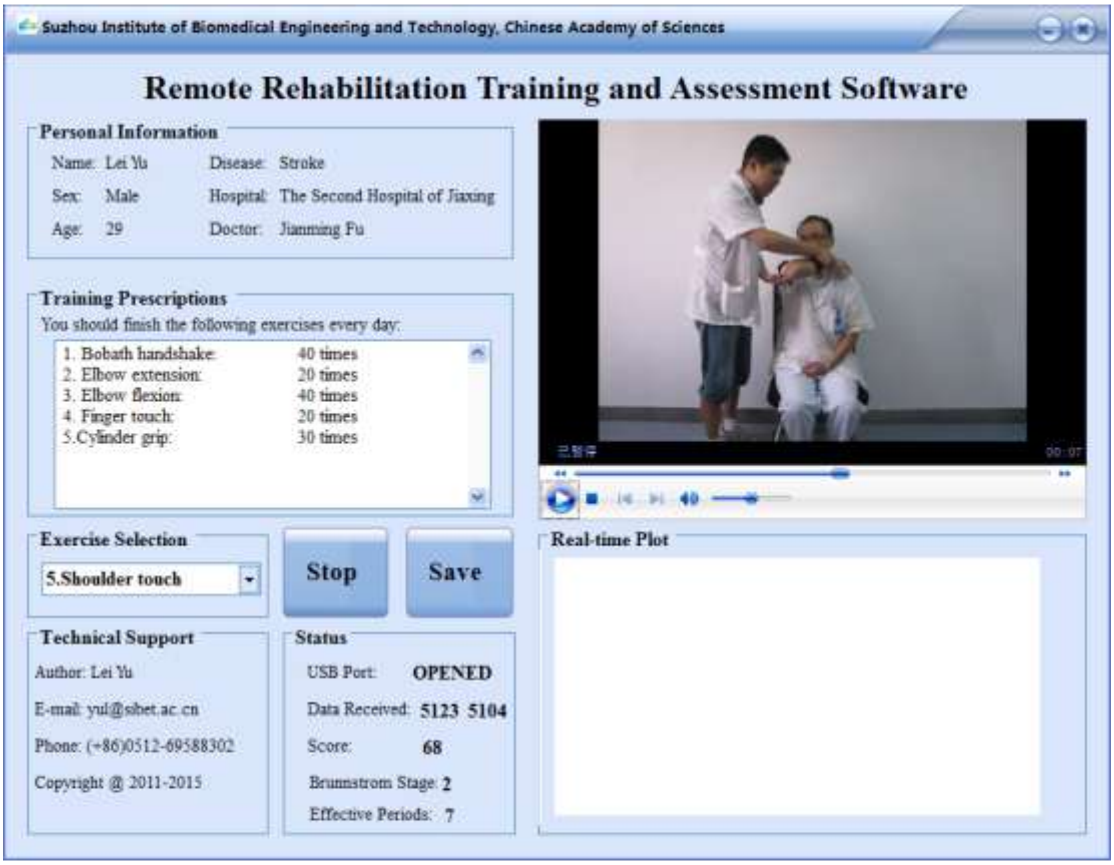

Figure 2 Automatic Brunnstrom stage classification and scoring
